# Ischemic stroke due to intracranial arterial dolichoectasia coexisting with spontaneous dissection of the basilar artery: A case report

**DOI:** 10.1097/MD.0000000000008422

**Published:** 2017-11-03

**Authors:** Fang Yang, Hong Yue, Lin Wu, Xia Qin, Lili Shi, Wei Qu

**Affiliations:** a Department of Neurology; b Department of Public Health, Rizhao People's Hospital, Rizhao, China.

**Keywords:** basilar artery, dissection, dolichoectasia, ischemic stroke

## Abstract

**Introduction:**

We present a rarely seen case of cerebral infarction due to intracranial dolichoectasia coexisting with spontaneous dissection of the basilar artery. A definition of dolichoectasia, its pathology, and imaging findings, as well as the clinical management and prognosis are briefly reviewed.

**Conclusion:**

We discuss in general terms the diagnosis of basilar artery dissection and its probable relationship with the occurrence of dolichoectasia.

## 1 Introduction

Intracranial arterial dolichoectasia (IADE) is an uncommon vascular disease defined as any pathological increase in length and diameter of at least 1 intracranial artery. It is present in approximately 12% of stroke patients, and most frequently involves the posterior circulation, a condition which is specifically known as vertebrobasilar dolichoectasia (VBD).^[1]^ The incidence of VBD is not entirely clear, but it has been estimated to occur in 1.3% of the adult population.^[2]^ Although most patients with IADE are asymptomatic, its occurrence increases the risk for a range of serious disorders, including cerebral infarction, cerebral hemorrhage, as well as obstructive ventricular hydrocephalus and compression of the cranial nerve or brainstem. Among the possible adverse events that arise from IADE, the most frequent is cerebral infarction.^[3,4]^ The occurrence of dolichoectasia in association with basilar artery dissection is very rare. Indeed, no such case has been reported in China to date, and a systematic search on the PubMed database yielded only a single report worldwide.^[5]^ We now describe a patient diagnosed with cerebral infarction, VBD, and basilar artery dissection.

## 2 Case report

Written informed consent was obtained from individual subjects and the experimental protocol was approved by the Ethics Committee of Rizhao People's Hospital.

**Figure 1:**
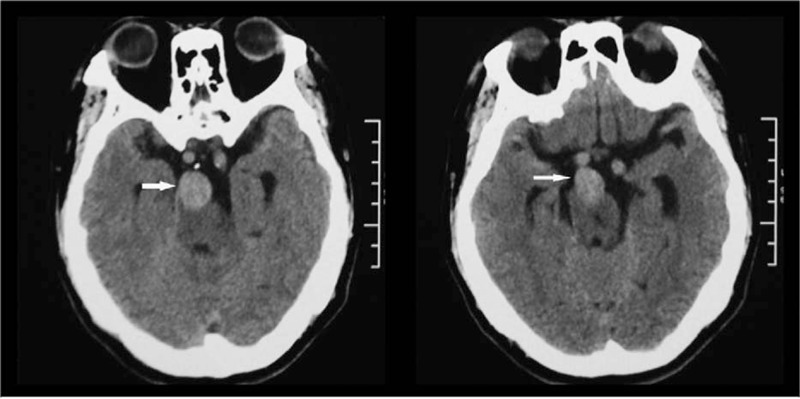
High-intensity signals on brain computed tomography.

**Figure 2:**
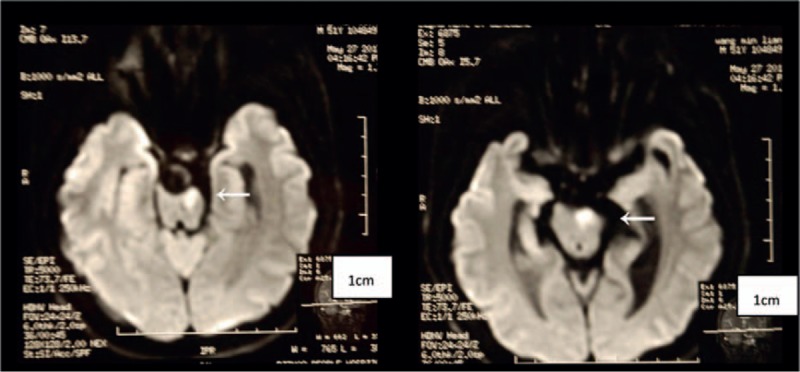
High-intensity signals on DWI indicating an acute pontine infarction.

**Figure 3:**
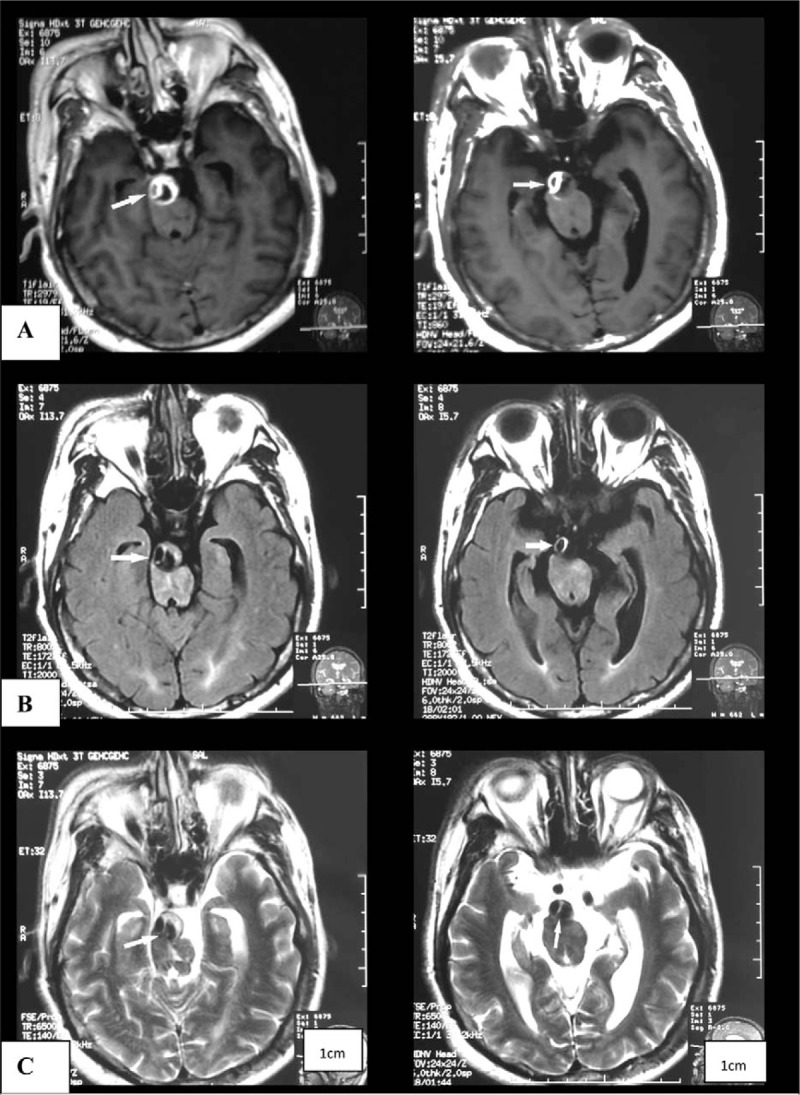
“Two lumen” sign on brain MRI, 1 is in crescent shape: (A) T1 flair; (B) T2 flair; and (C) weighted imaging. MRI = magnetic resonance imaging.

**Figure 4:**
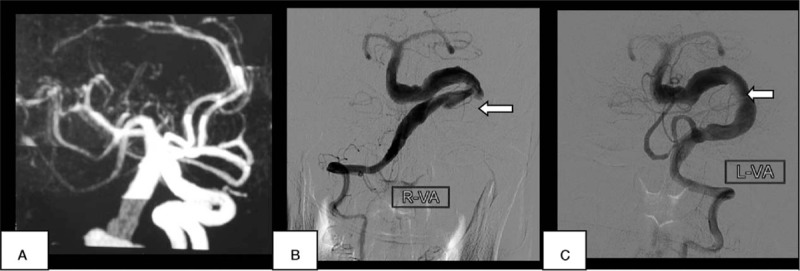
Vertebrobasilar dolichoectasia on MRA (A) and conventional angiography (B, C). Maximum basilar diameter = 8 mm. MRA = magnetic resonance angiography.

A 51-year-old right-handed man with a history of smoking, chronic nephritis, and hypertension was admitted to our hospital due to a sudden occurrence of right-sided weakness and dysarthria. Blood pressure was 158/94 mm Hg upon admission. Brain computed tomography (CT, General Electric, 64 rows of 128 layers) revealed some high-intensity signals at the pontine-medullary junction (Fig. 1). Magnetic resonance imaging (MRI, General Electric Signa HDxt 3.0T) indicated an acute pontine infarction, with presence of a “two-lumen” sign in the anatomic cross section (Figs. 2 and 3). Both conventional angiography and magnetic resonance angiography (MRA) confirmed the presence of VBD (Fig. 4). The patient had elevated serum creatinine and blood urea. Other laboratory investigations were positive for the presence of elevated urinary protein and hematuria, and a renal cyst was detected by renal ultrasound. The patient was diagnosed with cerebral infarction, VBD, basilar artery dissection, chronic nephritis, and hypertension. He was treated as an inpatient with aspirin, low molecular weight heparin (LMWH), and pravastatin. After 13 days, he was discharged with a significant improvement in muscle strength, which increased from grade 3 to grade 5, as assessed by the Medical Research Council grading scale of muscle power.

The patient was prescribed aspirin and pravastatin to prevent further infarct events. Two years after discharge, the patient's dysphagia and hemiplegia had gradually worsened to such an extent that he was unable to return to work. However, he refused to undergo further brain MRI and MRA investigations.

## 3 Discussion

IADE, also known as dilatative arteriopathy, is an angiopathy that is characterized by the notable dilation, elongation, and tortuosity of cranial arteries. The basilar artery has been proven to be affected in 80% of IADE patients with stroke.^[6,7]^ Despite this frequent association, there have been no received diagnostic criteria for diagnosing IADE to date. According to Greenfield's Pathology, a basilar artery lumen >4.5 mm is considered to be ectatic.^[5]^ Although some authors have suggested scaling arterial diameters to the total cranial volume, this revised threshold seems to have poor accuracy for defining IADE.^[8,9]^ Further studies are needed to define the cutoff between normal and pathological dilatation and elongation, taking into proper account the patient's age, gender, and possible variants in the circle of Willis morphology. At present, the Greenfield criterion of a basilar artery luminal diameter >4.5 mm on MRI axial sections seems to be the most commonly accepted criteria for cases that involve the posterior circulation.^[1]^ In our case report, the lumen of the basilar artery was measured at 8 mm, which easily meets the usual criteria.

We note that no validated diagnostic criteria have yet emerged for IADE of the anterior vasculature. IADE is usually seen in males >40 years old, whereupon its incidence increases with greater age. Since most IADE patients are asymptomatic, it may be most frequently encountered as an incidental finding in imaging studies. Consequently, there is a lack of high-quality population-based estimates of its incidence and prognosis. A study in Japan revealed that among people undergoing routine MRI and MRA examinations, the incidence of asymptomatic VBD was 1.3%^[2]^; but this reported incidence can greatly vary between different populations, depending on the age group, diagnostic criteria, and imaging methods employed.

The pathological processes leading to dolichoectasia involves changes in the extracellular matrix and smooth muscle cells, which are components of the tunica media.^[1]^ The integrity of the extracellular matrix can be affected by known vascular risk factors such as age, smoking, male gender, hypertension and coronary artery disease, or more specifically by an imbalance of matrix metalloproteinase (MMP) enzymes.^[1]^ The vascular matrix can also be affected in cases of Marfan syndrome or tortuosity syndrome, due to abnormal properties of the collagen in elastic fibers. In addition, IADE is associated with Fabry disease and Pompe disease in which smooth muscle cells are weakened due to defective lysosomal storage. Other associations of IADE include infection with HIV, autosomal dominant polycystic kidney, and abdominal aortic and other types of arterial ectasia. In pathological investigations, Masson trichrome staining shows rarefaction of elastic tissue in the tunica media with fragmentation of the internal elastic lamina in cases with known vascular risk factors, whereas patients with particular metabolic or genetic disorders have specific pathological findings.^[1]^ Furthermore, anatomical and hemodynamic factors may play a role in triggering dolichoectatic processes.^[10–12]^ Two studies have reported an association between anatomical variations in the circle of Willis and IADE.^[10,12]^ Thus, the vascular risk factors in our case (ie, male gender, hypertension, and smoking) likely impaired the extracellular matrix of the tunica media, and hemodynamic changes caused by hypertension facilitated the generation of dolichoectasia.

Although most patients with IADE are asymptomatic, ischemic stroke is the most common presentation in those with obvious clinical manifestations. However, other cases can present with compression on the cranial nerves or brain stem, obstructive hydrocephalus, intracranial hemorrhage, and subarachnoid hemorrhage. Potential mechanisms of ischemic stroke in patients with IADE include major distortion and obstruction of perforating arteries arising from the arteria with dolichoectasia, in situ thrombosis, and emboli from the dolichoectatic artery.^[13]^ Indeed, abnormalities of perforating arteries may be the most important mechanism, as patients with IADE are more likely to have lacunar infarction than other stroke patients. In our case, brain MRI revealed a paramedian pontine infarction, which may have been caused by occlusion of the perforating paramedian pontine artery. Deterioration of neurological state at follow-up after 2 years likely resulted from the progressive compression of the brain stem, but we cannot be certain of this due to lack of follow-up imaging data.

As noted above, the diagnosis of IADE is based on the luminal dilation in vascular images, although there is no widely accepted standard for optimal imaging modality or diagnostic criteria. In an important study using MR angiography, ectasia of the vertebrobasilar system was defined as an arterial diameter >4.5 mm in any location along its course. For the basilar artery, a length exceeding 29.5 mm or any lateral deviation >10 mm perpendicular to a straight line joining the BA origin to its bifurcation to MRA was considered abnormal. A vertebral artery with a length >23.5 mm to MRA >23.5 mm has been considered elongated.^[14]^ In general, digital subtraction cerebral angiography has been the gold standard for the diagnosis of cerebrovascular diseases. However, it is rarely used for cases of IADE because it shows only the dilated lumen, and not the pathology of the arterial wall.^[15]^ In addition, some investigators have reported an unacceptably high risk of transient or definite posterior circulation ischemia during and after conventional angiography.^[9]^ At present, brain MRI combined with MRA and high-resolution structural MRI appear to be the best methods for diagnosis. These methods can provide information on the integrity of the arterial wall, as well as the anatomical association between the dolichoectatic artery and its surrounding structures. Furthermore, MRI and MRA are recommended for follow-up,^[1]^ which could not be obtained in this case.

In our case, initial MRI revealed a “double lumen” of the basilar artery to T2-weighted imaging. This defect was deemed to be a dissection, which is defined as any tear in the wall of a major artery leading to the intrusion of blood within the layers of the arterial wall (intramural hematoma). Conventional angiography has been considered the standard of reference for the diagnosis of dissection, but it failed to reveal the dissection shown on MRI in our case. Intramural hematoma of intracranial dissection usually lies between the media and adventitia, leading to a dilation of the artery, instead of stenosis.^[16]^ Thus, there were no string-of-pearls sign, which often serves as an imaging-based indicator for luminal stenosis and dissection. We assume that a rupture within the connective tissue and *vasa vasorum* of the media was the most probable initial event, and that the intima was not affected, as such that catheter angiography did not reveal usual pathognomonic signs such as the “double lumen” or “intimal flap”. Thus, conventional angiography may be losing its gold standard status for this diagnosis due to the lack of visualization of arterial wall defects. Image quality from high-resolution structural MR now approaches that of catheter angiography. In addition, MRI can also reveal the intramural hematoma itself. Although a double lumen can also be seen in cases of fenestration, this differential diagnosis could be excluded in the present case by MRA and catheter angiography results; the diagnosis of basilar artery dissection could be obtained by MR alone. As basilar artery dissection is very rare in living patients, there have been no earlier reported cases of IADE coexisting with basilar artery dissection. We cannot be certain whether these 2 pathologies are directly related, but propose that the structural defect of the arterial wall may be the common underlying pathology.

For the IADE itself, no specific treatment is available to prevent further arterial dilation or elongation. Consequently, the management of patients with IADE mainly depends on their clinical symptoms. For patients with ischemic stroke, antiplatelet or anticoagulant treatment may be indicated, although its efficacy and safety remain unproven. Antiplatelet treatments are preferred for recurrent cerebral ischemia, as anticoagulants are thought to increase the risk of hemorrhagic events.^[17,18]^ Patients with high risk of rupture (basilar artery diameter >10 mm), should be prescribed antiplatelet treatments with caution, and strict blood pressure control is essential.^[19]^ The patient in our case used aspirin for secondary prevention of ischemic stroke, which has been proven effective at follow-up after 2 years. Low-molecular heparin was applied for 13 days prior to discharge, given that reduced blood flow in the ectatic basilar artery could then be seen. Brain MRI and MRA at 6 months and annually thereafter, or in case of new symptoms, are recommended for such cases. If there is further enlargement (>2 mm) or if the basal artery luminal diameter is much greater than 10 mm, surgery or endovascular procedures can be considered.^[1]^

Prognosis for IADE varies considerably due to the heterogeneity of the condition in terms of disease severity, study design, and clinical manifestation, although the magnitude of the basilar diameter is generally assumed to have an association with prognosis.^[1]^ As noted previously, patients with stroke and IADE often have lacunar infarctions, which generally have a better prognosis than the more extensive cardioembolic or atherothrombotic brain infarctions. Nonetheless, 1 study revealed that the prognosis was similar in patients with and without IADE.^[20]^

## 4 Conclusions

Although the case we presented is extreme, or perhaps unique, we hope that the accumulation of more clinical data would eventually better elucidate the relationship between IADE and basilar artery dissection. Due to advances in imaging, IADE is increasingly being noticed as a clinical syndrome, but high-quality prospective imaging studies promise to provide useful information for clinical application.
